# Automated production of recombinant human proteins as resource for proteome research

**DOI:** 10.1186/1477-5956-6-4

**Published:** 2008-01-28

**Authors:** Thorsten Kohl, Christian Schmidt, Stefan Wiemann, Annemarie Poustka, Ulrike Korf

**Affiliations:** 1 Division of Molecular Genome Analysis, German Cancer Research Center, Heidelberg, Germany

## Abstract

**Background:**

An arbitrary set of 96 human proteins was selected and tested to set-up a fully automated protein production strategy, covering all steps from DNA preparation to protein purification and analysis. The target proteins are encoded by functionally uncharacterized open reading frames (ORF) identified by the German cDNA consortium. Fusion proteins were produced in *E. coli *with four different fusion tags and tested in five different purification strategies depending on the respective fusion tag. The automated strategy relies on standard liquid handling and clone picking equipment.

**Results:**

A robust automated strategy for the production of recombinant human proteins in *E. coli *was established based on a set of four different protein expression vectors resulting in NusA/His, MBP/His, GST and His-tagged proteins. The yield of soluble fusion protein was correlated with the induction temperature and the respective fusion tag. NusA/His and MBP/His fusion proteins are best expressed at low temperature (25°C), whereas the yield of soluble GST fusion proteins was higher when protein expression was induced at elevated temperature. In contrast, the induction of soluble His-tagged fusion proteins was independent of the temperature. Amylose was not found useful for affinity-purification of MBP/His fusion proteins in a high-throughput setting, and metal chelating chromatography is recommended instead.

**Conclusion:**

Soluble fusion proteins can be produced in *E. coli *in sufficient qualities and μg/ml culture quantities for downstream applications like microarray-based assays, and studies on protein-protein interactions employing a fully automated protein expression and purification strategy. Future applications might include the optimization of experimental conditions for the large-scale production of soluble recombinant proteins from libraries of open reading frames.

## Background

A number of cDNA projects [1-4] and ORF cloning projects [5-9] currently provide comprehensive resources for functional analysis in various organisms comprising bacteria, plants, nematodes, as well as different mammalian species. However, a considerable number of identified proteins still lacks functional annotation. Protein microarrays present a promising tool among other approaches for the functional characterization of not yet annotated proteins [10-14]. In the recent past, microarray-based assays have been employed to identify novel protein-protein interactions, small molecule ligands, and protein phosphorylation sites [15,16]. The production of protein microarrays requires recombinant proteins in sufficient quantities and of adequate purity, or their production *in situ *[17]. In order to guarantee that proteins are full-length and presented in a defined concentration on the array, proteins must be produced ahead of the printing process. The baculovirus as well as yeast expression systems have been exploited to produce proteins on a large scale for subsequent production of microarrays [18]. Both expression systems introduce host-specific post-translational modifications. In contrast, the bacterial expression system *Escherichia coli *[19] produces proteins devoid of those post-translational modifications typically present in endogenously expressed mammalian proteins. This circumstance can be advantageous for certain applications, e.g. to screen for novel substrates of human kinases. Furthermore, *E. coli *is a well established expression system with known growth kinetics, robust handling characteristics, and high yields of recombinant proteins. Therefore, we selected *E. coli *as expression system for the automated production of uncharacterized human proteins from the LIFEdb database [20]. Hence, the resulting *in-vitro *data could help to bridge the knowledge from different large-scale technologies for functional genomics and proteomics applications [21,22].

**Figure 1:**
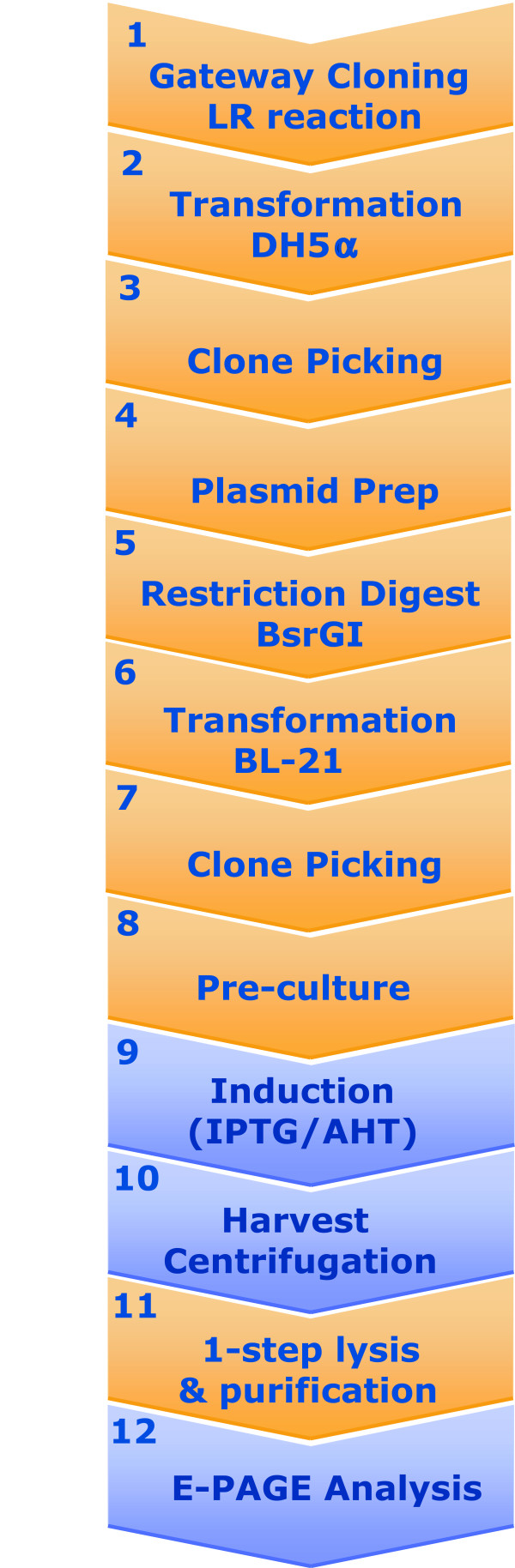
**Work flow of the automated protein production strategy**. Automated steps are shown in orange, steps involving manual intervention are shown in blue.

Different automated strategies are commercially available for bacterial high-throughput protein expression screening [23], or were established by different research groups [24-29]. These approaches have several drawbacks in common. For example, only a limited number of steps of the workflow are automated, leaving the challenge to integrate them into a fully automated system. The development of an automated platform for bacterial protein expression should also include DNA handling and quality control steps, as well as the production, purification and analysis of the recombinant proteins. Hence, we undertook an independent approach based on commercial robotics to set-up an improved platform for automated protein expression screening. All individual steps, including the preparation and characterization of expression clones, transformation into bacteria, picking of expression clones, growing bacterial cultures, induction of protein expression, harvesting raw protein extracts, protein affinity purification and subsequent quality control of purified proteins (Figure 1, Table 1) were performed in a multi-titer plate format and integrated in our protein production strategy. In addition, quality control steps were also included into the automated workflow. The correct insert size of the expression clones was verified by agarose gel electrophoresis, and the E-PAGE system (Invitrogen) was used to control the size and purity of affinity-purified proteins. This resulted in the development of a robust procedure which can easily be established on comparable clone picking and liquid handling equipment.

**Table 1:** Overview on instrumentation and consumables

	**Robot**	**Periphery**	**Consumables**
**Gateway cloning**	MultiProbe II EX, PerkinElmer, Wellesley	VortexGenie 2, Scientific Industries, Bohemia GeneAmp PCR System 9700, Applied Biosystems	Thermo-Fast 96 (ABgene, Epsom)
**Transformation**	MultiProbe II EX, PerkinElmer, Wellesley	GeneAmp PCR System 9700, Applied Biosystems	Thermo-Fast 96 (ABgene, Epsom) QTray, Vented (Genetix, New Milton)
**Clone Picking**	QPix (Genetix, New Milton)		6.0 mL Storage Plate with 48 Wells (ABgene)
**Plasmid purification**	MultiProbe II EX, PerkinElmer, Wellesley	Teleshake (H+P Labortechnik, München) Vacuum Manifold for MultiScreen Plates (Millipore)	2.2 mL Storage Plate (ABgene) Montage Plasmid Miniprep96 Kit (Millipore)
**Restriction digest**	MultiProbe II EX, PerkinElmer, Wellesley		Thermo-Fast 96 (ABgene, Epsom)
**Affinity purification**	MultiProbe II EX, PerkinElmer, Wellesley		ProtinoTrap M96/20 μm/L (M&N, Düren)

Our integrated automated approach for the production of recombinant human proteins [4,20] relies on the protein expression vectors previously described [30]. Accordingly, the four different expression vectors result in proteins N-terminally tagged with Glutathione-S-transferase- (GST) [31], hexahistidine- (His) [32], Maltose-binding protein- (MBP)/hexahistidine-tag [32], or hexahistidine and *E. coli *transcription-anti-termination-factor- (NusA) [33] (Table 2). In total, 96 Entry clones from the LIFEdb data base [20] encoding uncharacterized human proteins were selected for Gateway cloning [34] to yield expression clones required for the induction of protein expression [Additional file 1].

**Table 2:** Protein expression vectors [43]

**Vector**	**pQE80L-D33**	**pGST-1**	**pNusA**	**pMBP**
**Backbone**	pQE-80L	pGEX-6p-1	pASK75	pBAT4
**Tag 1**	His	GST	NusA	MBP
**Tag 2**	none	none	His	His
**Promoter**	T5/*lac*	tac	tetR	T7/*lac*
**Induction**	IPTG	IPTG	AHT	IPTG

## Results

### Technical set-up of the fully automated system

**Figure 2:**
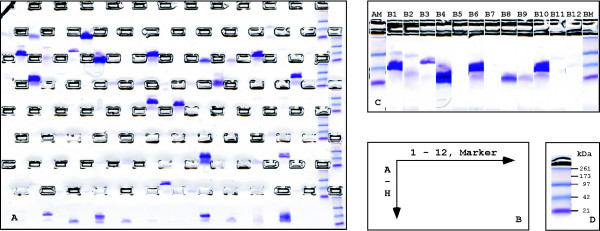
**Quality control of recombinant fusion proteins**. (A) Image of a Coomassie-stained E-PAGE gel, here shown for the purification of GST fusion proteins. (B) 96 samples can be loaded on a single E-PAGE gel comprising twelve lanes in eight rows (A-H). A single additional lane is available per row to accommodate a molecular weight standard. (C) Single lanes (each 2 cm in length) are assembled to an artificial gel image to facilitate sample analysis. (D) Example molecular weight marker separated by the E-PAGE system.

The liquid handling steps required for ORF cloning, protein expression and protein purification were implemented on the MULTI-probe II robot which was controlled with the application system software, if possible. Additional external equipment integrated into the robotic platform was navigated with the LabVIEW software. Clone picking was realized on the QPix robot. Figure 1 summarizes the single steps implemented into the automated routine. Open reading frames were transferred by Gateway LR reaction into four different destination vectors (Step1) and subsequently transformed into the bacterial strain DH5α for the amplification of recombinant expression plasmids (Step2). The automated restriction digest of expression plasmids confirmed the correct insert size for 361 of the 384 expression clones (Steps 3–5). Thus, 94% of destination clones were available for transformation into the bacterial strain BL21-SI (Step 6). In summary, each candidate was subjected to 15 different expression tests varying in the choice of fusion tag, induction temperature and purification strategy, or a combination thereof. Again, clone picking and the growth of pre-cultures were performed using our automated set-up (Steps 7, 8). However, the induction of protein expression by addition of IPTG or AHT is faster when performed manually (Step 9). Cultures were placed on a shaker at the indicated temperature (Step 10). Protein expression was stopped by removing the culture medium using gravity-driven filter plates. After lysis and affinity-purification (Step 11) the yield of recombination fusion proteins was analyzed using the E-PAGE system, a gel-based approach suitable for the high throughput analysis of proteins (Step 12). A single E-PAGE gel can accommodate all samples from a 96-well plate and additional molecular weight standards (Figure 2A, B). The final analysis is assisted by the E-PAGE software allowing to reassemble twelve sample lanes, corresponding to a single 96-well row, into a single image (Figure 2C). Calculation of the molecular weight of the purified fusion proteins is based on a molecular weight marker (Figure 2B, D). The yield is summarized in the Additional file 1. In order to count as successfully purified, the resulting fusion protein had to yield a clean band of the expected molecular weight. This analysis was performed using the E-PAGE system which separates proteins over a distance of merely 2 cm. The low resolution capacity of the E-PAGE system was accounted for by introducing the rule that only those proteins were regarded as successfully purified when at least two independent expression tests resulted in a protein band of the expected size. According to these criteria, 52% of the uncharacterized proteins were purified in fusion with at least one of the different tags, and quantities up to 10 μg/ml culture were obtained (Additional file 1). This yield was also reported for other strategies relying on the affinity purification of fusion proteins from small volume cultures [25,35]. However, the yield differs from our manual approach, where close to 80% of fusion proteins were obtained in quantities up to 100 μg/ml. Since the proteins analyzed in these two studies were comparable with respect to molecular weight and intracellular localization, we conclude that parameters such as aeration of culture, and the simplified one-step cell lysis and affinity purification strategy contribute to the reduced overall yield of the automated protein production strategy.

### Influence of Fusion Tag and Temperature on Protein Yield

**Figure 3:**
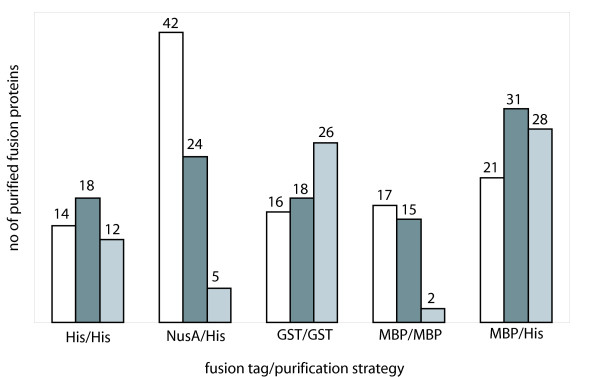
**Influence of fusion tag and induction temperature on fusion protein yield**. Successfully purified human fusion proteins sorted according to fusion tag and purification strategy. Protein expression was induced at 25°C (white), 30°C (dark grey) and 37°C (light grey), respectively.

The influence of the different fusion tags was examined (Figure 3) and compared with the outcome of our manual approach. With respect to the impact of the induction temperature on His-tagged protein expression, 15% (14 proteins), 19% (18 proteins), 5% (5 proteins) of His-tag proteins were purified when induced at a temperature of 25°C, 30°C, and 37°C, respectively. For reasons of technical simplicity, a one-step lysis and purification procedure was performed in the automated approach. This one-step procedure monitored exclusively the successfully purified proteins without analyzing the percentage of inducible proteins. Moreover, with an average yield of close to 30%, His-tagged fusion proteins were slightly better soluble when protein expression was induced in the manual approach [30].

We could confirm for the automated approach that the NusA tag potentially increases the solubility of difficult to express proteins. The expression of NusA-fusion proteins is more efficient at lower temperature [30]. For example, 42 (44%) NusA-fusion proteins could be purified when protein expression was induced at 25°C, but only 24 (25%) and 5 (5%) of NusA fusion proteins were purified when protein expression was induced at 30°C and 37°C, respectively. Quite the reverse was found for GST fusion proteins which were produced more efficiently when protein expression was induced at elevated temperature. In our automated approach, 26 GST-fusion proteins (27%) were successfully purified when protein expression was induced at 37°C, 18 (19%) at 25°C, and 16 (17%) at 20°C. The MBP-tag behaved comparably to the NusA-tag, the number of successfully purified proteins decreased with increasing induction temperature (17, 15, and 2 proteins with increasing induction temperature).

Furthermore, we could confirm that amylose-based affinity chromatography does not perform well in an automated setting previously reported by Braun *et al*. [25]. In detail, MBP/His-fusion protein purified by metal chelate chromatography resulted in 36 soluble fusion proteins (38%) whereas merely 19% of MBP/His fusion tag proteins were obtained after amylose-based affinity chromatography (Table 3).

**Table 3:** Yield of soluble recombinant protein. Results sorted according to ORF size [kDa].

**ORF size [kDa]**	**ORF count [n]**	**purif. ORF [n]**	**Yield ORF [%]**	**His^a ^His^b^**	**NusA^a ^His^b^**	**GST^a ^GST^b^**	**MBP^a ^MBP^b^**	**MBP^a ^His^b^**
**0–9**	2	1	50	1	0	0	0	1
**10–19**	10	9	90	4	8	8	5	7
**20–29**	21	15	70	6	11	8	6	14
**30–39**	15	10	70	2	8	4	3	6
**40–49**	15	10	70	3	10	6	4	7
**50–59**	3	1	30	0	1	0	0	0
**60–69**	8	3	40	2	1	0	0	0
**70–79**	5	2	40	1	1	0	0	0
**80–89**	7	2	30	0	2	0	0	1
**90–99**	6	0	0	0	0	0	0	0
**≥ 100**	4	0	0	0	0	0	0	0

^a ^fusion tag

^b ^tag employed for purification strategy

## Discussion

### Development of the automated process

A comprehensive automation of working steps including transformation, bacterial culture, cell disruption and protein extraction, as well as protein purification, and quality control of the purified proteins has been developed to provide material for the large-scale *in vitro *characterization of human proteins. Every single step (Figure 1) contributed its own particular challenge which had to be solved to fit into a comprehensive automated protein expression approach.

Bacteria can efficiently be transformed by electroporation on a single-clone basis. However, this procedure is difficult to automate and to parallelize, and technical limitations exclude its application in a multi-well format. Therefore the transformation of bacteria by heat shock was chosen, which can proficiently be realized by integrating a PCR machine or a thermoblock on the robot desk.

The vessel dimensions, such as fermenter, Erlenmeyer flask, tube and deep well block, as well as well shape, size and volume and the shaking frequency influence the gas-liquid mass transfer characteristics. Gas-liquid mass transfer phenomena in microtiter plates were described by Hermann et al. [36], and therefore 48-well blocks instead of 96-well blocks were chosen to insure sufficient aeration of the cultures. When we compared bacterial growth rates in 48-well plates with differently shaped wells, we observed that the cultures grew at a higher rate when square-shaped flat bottom wells were employed instead of wells with a round well U-bottom. This reflects most likely the more vigorous mixing of liquids in square-shaped wells. In the automated set-up presented here, bacterial cell lysis and affinity chromatography were performed as a one-step procedure without relying on sonication to break up cell walls. Insoluble material was not separated from the slurry due to difficulties to implement this step in our automated platform. Consequently, this automated strategy does not deliver information regarding the induction of insoluble fusion proteins.

### Influence of fusion tag and induction temperature on protein induction

Hydrophilic fusion tags such as NusA, MBP and GST enhance fusion protein solubility [33] when fused N-terminally to the ORF. This has previously been tested in large-scale protein expression strategies [25,30]. In the case of NusA and MBP fusion tags, protein expression at low temperatures yielded a higher percentage of soluble recombinant proteins. According to results from our automated approach, this finding applies exclusively to proteins induced at a low level (i.e. ORFs no. 3, 6, 96). In contrast, proteins inducible with a high yield were found to remain soluble over a broad temperature range (i.e. ORF no. 13, 18, 22, 26, 41, 79).

The MBP-tag is known to support proper folding of recombinant proteins and to enhance protein solubility [37,38]. The affinity of MBP to amylose can be exploited for affinity purification. Nevertheless, the binding of MBP to amylose is too inefficient to be useful in a high-throughput setting, and a high proportion of MBP fusion proteins were observed in the flow through and wash fractions, resulting in a low overall yield. Thus, purifying MBP-fusion proteins via their internal His-tag on metal chelating chromatography turned out to be the better choice. With respect to difficult-to-express proteins such as membrane proteins, the NusA tag is useful as long as the induction of protein expression is performed at 20–25°C, and with sufficient aeration [30].

### Characterization of fusion proteins

Occasionally, translation of GST- and MBP-tag fusion proteins stopped prematurely and the fusion tag itself co-purified with the fusion protein. This effect was even more pronounced for the NusA-tag. In summary, controlling quality and purity of purified recombinant proteins by SDS-PAGE, for example by using the E-PAGE system, is mandatory as efficient quality control.

### Comparison with other approaches

Bussow and coworkers have described the heterologous high-throughput production of 10,825 human clones in *E. coli*. In this case, 1,866 proteins purified as hexahistidine-tagged soluble protein of at least 15 kDa (17%) [39]. A comparable success rate, 16 % of soluble His-tagged proteins, was obtained in this approach with respect to the automated purification of His-tagged fusion proteins. However, in contrast to their approach, the vacuum-filter plate was replaced with a gravity-filter plate in our set-up, thus reducing extensive foaming that we observed in filtration steps after applying a strong vacuum. Extensive foam formation can easily result in well-to-well cross contamination.

Braun *et al*. [25] tested the automated purification of 32 different human proteins sizing between 16–220 kDa using four different fusion tags, among them MBP, GST and the hexahistidine tag. According to their results, sixty percent of the proteins were purified under non denaturing conditions. MBP and GST fusion tag proteins resulted in better yields than fusion proteins with a short tag, such as the hexahistidine tag. They also reported that the affinity of MBP to amylose as too low to be employed in a high throughput strategy. In contrast, 21% of GST fusion proteins and 11% of MBP fusion protein were purified, when expression tests performed at the three different temperatures were taken into account. However, Braun et al. tested protein expression exclusively at 25°C, and the apparent discrepancy between their results and our results can be explained with the temperature dependence of GST fusion protein expression. In our high-throughput set-up, the best yield was obtained when GST fusion proteins were induced at 37°C. Moreover, when our 37°C data were omitted from the comparison, success rates for our data set and for the Braun study were comparable. Pryor and Leiting tested the efficiency of the GST tag and the MBP tag for the production of soluble recombinant protein on a small scale at two different induction temperatures, 18°C and 37°C, and reported the MBP tag as superior at both temperatures [40]. This result contrasts our experience with the MBP fusion tag, but might be explained with by the very limited number of only two proteins tested by Pryor and Leiting.

Moreover, Braun et al. [25] observed that the yield of recombinant proteins also strongly depends on the subcellular localization of the endogenous protein. Integral membrane proteins and secreted proteins requiring separate optimization and purification methods and were therefore excluded from their study. As much as 50% of the total proteins encoded in the human genome are supposedly membrane or secreted proteins, and a unique strategy would be useful to purify also this large fraction of proteins. In contrast to Braun *et al*. [25], the strategy presented here did not exclude difficult to express proteins. We previously reported that the NusA tag is beneficial for the expression of difficult proteins which was confirmed in other non high throughput settings [24]. However, Hammarström *et al*. [41] compared the benefits of seven different fusion tags for the production of recombinant proteins in *E. coli*, and MBP was reported to be superior over NusA as fusion tag. In this instance, only small proteins (< 20 kDa) were tested, and protein expression was induced at 37°C. Again, the strong temperature dependence of both tags and the fact that only small proteins had been selected certainly contribute to the observed differences.

## Conclusion

The automated protein production approach presented here introduces a simplified one-step lysis and purification procedure for affinity purification of soluble mammalian proteins. According to our data, NusA fusion proteins should be induced at a low temperature (25°C), whereas GST fusion proteins are better induced at elevated temperature. The purification of fusion protein should be based on metal chelating chromatography, or on affinity to Glutathione. Our strategy can ideally be applied as screening routine for the identification of highly soluble proteins which are required in structural analysis. The selected target proteins can subsequently be produced on a larger scale using a manual approach. In addition, our automated strategy is also useful, when large numbers of different fusion proteins are required, but μg-quantities of purified proteins are sufficient. This applies to high-throughput approaches as realized in functional assays performed in the protein microarray format, or on arrays with compound libraries. In summary, a robust robotic set-up based on standard instrumentation is described which overcomes inefficient steps from other strategies by introducing optimized automated steps, and comprises a larger number of automated steps than before described. This set-up can easily be established on comparable liquid-handling robotics.

## Methods

### Automated cloning, purification and characterization of Gateway-expression clones

The Gateway Cloning system (Invitrogen, Karlsruhe, Germany) was used to generate the protein expression clones listed in the Additional file 1[34]. Open reading frames were available as entry clones without their native stop codons in vector pDONR201 [42]. Consequently, all fusion proteins contain C-terminally additional amino acids encoded by the respective destination plasmids [30]. All steps to clone the human ORFs [4,20]; e.g. LR-reaction, transformation into bacteria, plasmid purification, normalization of DNA concentration, were automated and carried out in a 96-well format. Pipetting was performed on a Perkin Elmer Multiprobe II robot. The LR-reaction was performed in a volume of 15 μl; 3 μL LR reaction buffer (5×), 150 ng expression vector (5 μL) and 2 μL LR CLONASE enzyme mix were pipetted into each well. Finally 5 μL (20 ng/μL) of entry clone DNA were added. Mixing was performed by shaking (Variomag Teleshake, H+P Labortechnik). The plate was transferred on to an integrated PCR machine (Applied Biosystems, Geneamp PCR System 9700), and incubated at 16°C over night. The reaction was stopped by addition of 5 μL Proteinase K (Invitrogen). Next, 50 μL of competent DH5α cells were pipetted into each well of a chilled 96-well plate. 5 μL LR-reaction were added to each of the wells. For heat shock transformation, the plate was placed manually on to a PCR machine, and the samples were incubated at 42°C for 45 s, then the temperature adjusted to 0°C and incubation continued for another 5 min. Finally 500 μL of prewarmed LB medium were added, and the plate was placed for 1 h onto an orbital shaker (Infors) at 37°C. A suspension with transformed bacteria (100 μL) was pipetted from each well to a corresponding well of a 48-well agar plate (Genetix, New Milton, UK), containing 3–5 glass beads of 3 mm diameter (Roth). A homogenous distribution of the suspension was achieved by gentle shaking. Bacteria were grown over night at 37°C. Single clones were picked using the QPix robot (Genetics). Plasmids were prepared from single colonies using commercial kits (Montage 96, Plasmid MiniprepKit, Millipore), with the protocol adapted to a Perkin Elmer Multiprobe robot. Expression clones were confirmed by robotically performed restriction digestion with *Bsr*G1, cleaving the Gateway recombination sites, and electrophoresis in 96 lane agarose gels (1% agarose in TAE buffer). The concentration of DNA was estimated by a 260/280 measurement in Costar UV Plates (Corning Lifesciences, Acton) on a SpectraMax190 (Molecular Devices, Sunnyvale).

### Automated induction of protein expression

The heat shock transformation was performed using 50 ng of the expression plasmid added to 50 μL *E. coli *BL21(DE3) cells (Invitrogen). Target proteins were expressed in duplicate on a 4 mL scale in deep well blocks (Greiner).

Precultures were inoculated with a single colony and from a 48-well agar plate (Genetix QPix), and grown in 48 well blocks (Greiner) in 1 mL LB medium. After incubation for 16 h at 30°C, aliquots of 100 μL preculture were used to inoculate 3.6 mL prewarmed LB medium in the 48-deep well format. Two 48-well blocks were processed at a time at 25°C, 30°C, or 37°C. Recombinant protein expression was induced after 1.5 h, 2 h, and 3.5 h, depending on the expression temperature, by adding either 1 mM IPTG or 0.43 mM AHT (see Table 4 for details). Bacteria were harvested after 12 h continued culture by centrifugation for 10 min at 2,500 × g. Medium was removed by aspiration, and the remaining pellets were kept at -20°C for further analysis.

**Table 4:** Buffers and materials used for protein purification

	**His-Tag**	**GST-Tag**	**MBP-Tag**
**Resin**	Ni-NTA Superflow (QIAGEN)	Glutathione Sepharose 4B (Amersham Biosciences)	Amylose resin (New England Biolabs)
**Resuspension/extraction**	50 mM NaH_2_PO_4_, pH 8 300 mM NaCl 10 mM imidazole adjust pH w/NaOH	20 mM Tris-HCl, pH 8 500 mM NaCl 1 mM EDTA 1 mM DTT	20 mM Tris-HCl, pH 7.4 200 mM NaCl 1 mM EDTA
**Wash**	50 mM NaH_2_PO_4_, pH 8 300 mM NaCl 20 mM imidazole adjust pH w/NaOH	PBS, pH 7.3	20 mM Tris-HCl, pH 7.4 200 mM NaCl 1 mM EDTA
**Elution**	50 mM NaH_2_PO_4_, pH 8 300 mM NaCl 250 mM imidazole adjust pH w/NaOH	PBS 20 mM glutathione (red.)	Wash buffer 10 mM maltose

The E-PAGE system of Invitrogen was utilized for protein expression analysis, where a single gel can be loaded with 96 samples. All samples from one induction were loaded on a single E-PAGE gel with the pipetting robot. Electrophoresis was controlled by the standard soft- and hardware of the robot (Multiprobe, Perkin Elmer).

### Automated protein purification and characterization of fusion proteins

Deep well blocks containing the frozen *E. coli *pellets were placed on a Variomag shaker that had been mounted on the operation deck of the Multiprobe II robot, and shaker movement was controlled through the LabVIEW software. The cell pellets were thawed on ice and resuspended in 500 μL resuspension buffer (see Table 4 for details, one tablet EDTA-free protease inhibitor (Roche) was added to 50 mL buffer). A 50 μL buffer aliquot containing 0.3 units/μL Benzonase (Merck), 2.6 μg/μL Lysozyme (Sigma), and 6.5 mM PMSF (Roth) was added. After mixing briefly, 100 μL of a 50 % slurry affinity resin were pipetted to each well, and incubated for 20 min at RT with shaking adjusted to 500 rpm. The slurry was transferred to a 20 μm gravity-driven filter plate (M96/20 μm/I, MACHEREY-NAGEL), and placed on a vacuum chamber (QIAGEN). The filtration was supported by a slight vacuum of 50 mbar for 20 s. The resin was washed three times with 450 μL of the appropriate buffer (Table 4) also supported by a slight vacuum. Finally, a microtiter plate was placed in the vacuum chamber and the target proteins were eluted in three steps using 80 μL elution buffer.

### Automated analysis of the purified fusion proteins

20 μL eluate were mixed with sample buffer and analyzed (E-PAGE system). 96 samples and appropriate markers were loaded and analyzed per gel. Gels were run at 500 V for 10 min, stained with 0.1% Coomassie R250, destained, and scanned for evaluation and documentation (Diana II Imaging System, raytest). The gels were analyzed manually and the resulting information was stored in an internal data base.

## Abbreviations

AHT (Anhydrotetracyclin), IPTG (Isopropyl-β-D-galactopyranoside), IMAC (immobilized metal affinity chromatography), LB (Luria-Bertani Medium), MBP (Maltose-binding protein) NusA (*E. coli *transcription-termination anti-termination factor).

## Competing interests

The author(s) declare that they have no competing interests.

## Authors' contributions

TK conceived the high-throughput screen of mammalian ORFs and tested different solutions to develop a high-throughput protein expression and purification strategy. In addition, he analyzed and summarized the results of the study.

CS programmed the robots to adapt the workflow to an automated process.

SW provided clones from the German cDNA consortium.

AP finally approved the manuscript.

UK contributed to the design of the study and drafted the manuscript.

All authors have read and approved the final manuscript.

## Supplementary Material

Additional file 1 Overview on Gateway Entry Clones and results from automated protein expression screening. The table provides Gateway entry clone information and a summary of the results from the automated protein expression screening and affinity purification for each individual fusion protein tested at three different temperatures. Click here for file
