# Supplementary material for: Automated production of recombinant human proteins as resource for proteome research
Source: Proteome Sci. 2008 Jan 28;6:4. doi: 10.1186/1477-5956-6-4 (PMC2266735; doi:10.1186/1477-5956-6-4)
Supplement: Additional file 1 — Overview on Gateway Entry Clones and results from automated protein expression screening. The table provides Gateway entry clone information and a summary of the results from the automated protein expression screening and affinity purification for each individual fusion protein tested at three different temperatures. [file 1477-5956-6-4-S1.doc]

| **Additional table 1: Overview on Gateway Entry Clones and results from automated protein expression screening** | | | | | | | | | | | | | | | | | | | | | | | |
| --- | --- | --- | --- | --- | --- | --- | --- | --- | --- | --- | --- | --- | --- | --- | --- | --- | --- | --- | --- | --- | --- | --- | --- |
| No | ORF ID^a^ | Accession No. ^b^ | Gene ID^c^ | Gene Symbol^c^ | Protein Name^d^ | His^e^ | | | NusA-His^e^ | | | GST^e^ | | | MBP^e^ | | | MBP-His^e^ | | | MW [kDa]^f^ | pI^g^ | Loc^h^ |
|  |  |  |  |  |  | 25 | 30 | 37 | 25 | 30 | 37 | 25 | 30 | 37 | 25 | 30 | 37 | 25 | 30 | 37 |  |  |  |
| 1 | DKFZp761A19121 | AL136545 | 80036 | TRPM3 | transient receptor potential cation channel | 1 | - | - | 2 | 1 | - | - | - | - | - | - | - | - | - | - | 36,8 | 10 | cy,nu |
| 2 | DKFZp761A19121 | AL136545 | 80036 | TRPM3 | transient receptor potential cation channel | 1 | - | - | 3 | 1 | - | - | - | - | - | - | - | 1 | - | - | 36,8 | 10 | cy,nu |
| 3 | DKFZp761I0112 | AL136556 | 57223 | SMEK2 | SMEK homolog 2, suppressor of mek1 (Dictyostelium) | - | - | - | 2 | 1 | - | - | - | - | - | - | - | - | - | - | 32,3 | 5 | ne |
| 4 | DKFZp761I0112 | AL136556 | 57223 | SMEK2 | SMEK homolog 2, suppressor of mek1 (Dictyostelium) | - | - | - | 2 | 1 | - | - | - | 1 | - | - | - | - | - | - | 32,3 | 5 | ne |
| 5 | DKFZp761I141 | AL136564 | 83746 | L3MBTL2 | l(3)mbt-like 2 (Drosophila) | 1 | 1 | - | - | - | - | - | - | - | - | - | - | - | - | - | 69,3 | 6 | nu |
| 6 | DKFZp761I141 | AL136564 | 83746 | L3MBTL2 | l(3)mbt-like 2 (Drosophila) | 2 | 1 | - | - | - | - | - | - | - | - | - | - | - | - | - | 69,3 | 6 | nu |
| 7 | DKFZp761G2023 | AL136570 | 26468 | LHX6 | LIM homeobox 6 | - | - | - | - | - | - | - | - | - | - | - | - | - | 1 | 1 | 40,0 | 8 | nu |
| 8 | DKFZp761J1923 | AL136574 | 57447 | NDRG2 | NDRG family member 2 | - | - | - | 1 | 2 | - | - | - | - | - | - | - | - | 1 | 1 | 40,8 | 4 | go |
| 9 | DKFZp761J1923 | AL136574 | 57447 | NDRG2 | NDRG family member 2 | - | 1 | - | 1 | 2 | - | - | - | - | - | - | - | - | 1 | 1 | 40,8 | 4 | go |
| 10 | DKFZp761K1524 | AL136581 | 64430 | C14orf135 | chromosome 14 open reading frame 135 | 1 | - | - | - | - | - | - | - | - | - | - | - | - | - | - | 32,5 | 5 | cy,nu |
| 11 | DKFZp761P2324 | AL136584 | 65009 | NDRG4 | NDRG family member 4 | - | - | - | 3 | 2 | - | - | - | 1 | - | - | - | - | 1 | - | 37,1 | 5 | go |
| 12 | DKFZp761C222 | AL136587 | 55326 | AGPAT5 | 1-acylglycerol-3-phosphate O-acyltransferase 5 (lysophosphatidic acid acyltransferase, epsilon) | - | - | - | - | - | - | - | - | - | - | - | - | - | - | - | 42,1 | 9 | mi |
| 13 | DKFZp761G122 | AL136591 | 51440 | HPCAL4 | hippocalcin like 4 | 3 | 3 | 3 | 3 | 2 | 2 | 3 | 3 | 3 | 2 | 2 | - | 3 | 3 | 3 | 22,2 | 4 | cy,nu |
| 14 | DKFZp564A2416 | AL110124 | 26010 | LOC26010 | viral DNA polymerase-transactivated protein 6 | - | - | - | - | - | - | - | - | - | - | - | - | - | - | - | 61,7 | 10 | cs,mt- |
| 15 | DKFZp564K1216 | AL049933 | 2770 | GNAI1 | guanine nucleotide binding protein (G protein), alpha inhibiting activity polypeptide 1 | - | - | - | 2 | - | - | 2 | 1 | 2 | - | - | - | - | 2 | - | 40,3 | 5 | go |
| 16 | DKFZp564K1216 | AL049933 | 2770 | GNAI1 | guanine nucleotide binding protein (G protein), alpha inhibiting activity polypeptide 1 | - | - | - | 3 | 2 | - | - | - | - | - | - | - | - | - | - | 40,3 | 5 | go |
| 17 | DKFZp564P1516 | AL110128 | 9374 | PPT2 | palmitoyl-protein thioesterase 2 | - | - | - | - | - | - | - | - | - | - | - | - | - | 1 | - | 34,9 | 6 | er |
| 18 | DKFZp564B2123 | AL136612 | 83988 | NCALD | neurocalcin delta | 3 | 3 | 3 | 3 | 2 | - | 3 | 3 | 3 | 1 | 1 | - | 3 | 3 | 2 | 22,2 | 5 | cy,nu |
| 19 | DKFZp564O0823 | AL080121 | 25849 | DKFZP564O0823 | DKFZP564O0823 protein | - | - | - | - | - | - | - | - | - | - | - | - | - | - | - | 25,0 | 4 | go |
| 20 | DKFZp564P1263 | AL136653 | 11067 | C10orf10 | chromosome 10 open reading frame 10 | - | - | - | - | - | - | - | - | - | - | - | - | - | - | - | 23,4 | 11 | uk |
| 21 | DKFZp564K0164 | AL117618 | 5162 | PDHB | pyruvate dehydrogenase (lipoamide) beta | - | - | - | - | - | - | - | - | - | - | - | - | - | - | - | 37,2 | 5 | cy |
| 22 | DKFZp564N1272 | AL136676 | 23710 | GABARAPL1 | GABA(A) receptor-associated protein like 1 | 3 | 3 | 3 | 3 | 2 | - | 3 | 3 | 3 | 2 | - | - | 2 | 3 | 3 | 14,0 | 9 | cy,nu |
| 23 | DKFZp564N2378 | AL136692 | 29086 | HSPC142 | HSPC142 protein | - | - | - | 2 | - | - | - | - | 1 | 2 | 2 | - | - | 2 | - | 36,6 | 4 | cy,nu |
| 24 | DKFZp564J157 | AL122042 | 54458 | PRR13 | proline rich 13 | - | - | - | 3 | 3 | - | - | 1 | 1 | - | 1 | - | 1 | 2 | 1 | 15,4 | 10 | cy,nu |
| 25 | DKFZp564J157 | AL122042 | 54458 | PRR13 | proline rich 13 | - | - | - | 3 | 3 | 2 | - | 1 | 1 | 2 | 2 | - | 1 | 3 | 2 | 15,4 | 10 | cy,nu |
| 26 | DKFZp564E2182 | AL050261 | 25842 | ASF1A | ASF1 anti-silencing function 1 homolog A (S. cerevisiae) | 3 | 3 | 3 | 2 | - | - | 3 | 2 | 3 | 2 | 2 | - | 2 | 3 | 3 | 23,0 | 4 | nu |
| 27 | DKFZp566J151 | AL136702 | 3417 | IDH1 | isocitrate dehydrogenase 1 (NADP+), soluble | - | - | - | 2 | - | - | - | - | 1 | 2 | 2 | - | 1 | 2 | 1 | 46,6 | 6 | cy |
| 28 | DKFZp566J151 | AL136702 | 3417 | IDH1 | isocitrate dehydrogenase 1 (NADP+), soluble | - | - | - | 1 | - | - | - | - | 1 | 2 | 2 | - | 1 | 2 | 2 | 46,6 | 6 | cy |
| 29 | DKFZp566E2324 | AL136708 | 79992 | C6orf59 | chromosome 6 open reading frame 59 | - | - | - | - | - | - | - | - | - | 2 | 2 | 2 | - | - | 1 | 20,9 | 6 | cy |
| 30 | DKFZp566N2024 | AL136709 | 51225 | ABI3 | ABI gene family, member 3 | - | - | - | - | - | - | - | - | - | - | - | - | - | - | - | 38,9 | 4 | cy |
| 31 | DKFZp566N2024 | AL136709 | 51225 | ABI3 | ABI gene family, member 3 | - | - | - | - | - | - | - | - | - | - | - | - | - | - | - | 38,9 | 4 | cy |
| 32 | DKFZp566N2024 | AL136709 | 51225 | ABI3 | ABI gene family, member 3 | - | - | - | - | - | - | - | - | - | - | - | - | - | - | - | 38,9 | 4 | cy |
| 33 | DKFZp566B023 | AL050273 | 25873 | RPL36 | ribosomal protein L36 | - | - | - | 3 | 2 | 2 | - | 2 | 2 | - | - | - | - | - | 2 | 12,3 | 12 | nu,nl |
| 34 | DKFZp566F123 | AL050276 | 26137 | ZBTB20 | zinc finger and BTB domain containing 20 | - | - | - | - | - | - | - | - | - | - | - | - | - | - | - | 73,4 | 6 | nu |
| 35 | DKFZp566F123 | AL050276 | 26137 | ZBTB20 | zinc finger and BTB domain containing 20 | - | - | - | - | - | - | - | - | - | - | - | - | - | - | - | 73,4 | 6 | nu |
| 36 | DKFZp566G223 | AL117424 | 25932 | CLIC4 | chloride intracellular channel 4 | 2 | - | - | 3 | 3 | - | 3 | 2 | 2 | - | - | - | 2 | 3 | 2 | 28,8 | 5 | cy,nu |
| 37 | DKFZp566I133 | AL136711 | 81671 | TMEM49 | transmembrane protein 49 | - | - | - | - | - | - | - | - | - | - | - | - | - | - | - | 46,3 | 6 | er |
| 38 | DKFZp566I133 | AL136711 | 81671 | TMEM49 | transmembrane protein 49 | - | - | - | - | - | - | - | - | - | - | - | - | - | - | - | 46,3 | 6 | er |
| 39 | DKFZp566K013 | AL136712 | 26052 | DNM3 | dynamin 3 | - | - | - | - | - | - | - | - | - | - | - | - | - | - | - | 97,1 | 8 | or |
| 40 | DKFZp566K013 | AL136712 | 26052 | DNM3 | dynamin 3 | - | - | - | - | - | - | - | - | - | - | - | - | - | - | - | 97,1 | 8 | or |
| 41 | DKFZp566A0646 | AL136715 | 79719 | FLJ11506 | hypothetical protein FLJ11506 | 3 | 3 | 3 | - | - | - | - | - | - | 2 | 2 | - | - | 1 | 1 | 34,5 | 4 | cy |
| 42 | DKFZp566L1246 | AL136722 | 30819 | KCNIP2 | Kv channel interacting protein 2 | 3 | 3 | 3 | 3 | 3 | - | 3 | 3 | 3 | 1 | 1 | - | 3 | 3 | 2 | 26,0 | 4 | cy,nu |
| 43 | DKFZp434H0311 | AL136742 | 649622 | LOC649622 | similar to thioredoxin domain-containing 2 | - | 1 | - | 3 | 3 | - | 3 | 1 | 3 | 3 | 3 | 2 | 2 | 3 | 1 | 12,7 | 4 | cy,nu |
| 44 | DKFZp434L0714 | AL136752 | 60684 | FLJ12716 | FLJ12716 protein | - | - | - | - | - | - | - | - | - | - | - | - | - | - | - | 91,8 | 6 | cy |
| 45 | DKFZp434L0714 | AL136752 | 60684 | FLJ12716 | FLJ12716 protein | - | - | - | - | - | - | - | - | - | - | - | - | - | - | - | 91,8 | 6 | cy |
| 46 | DKFZp434C2415 | AL136757 | 79876 | UBE1DC1 | ubiquitin-activating enzyme E1-domain containing 1 | - | - | - | 2 | - | - | - | - | 1 | 1 | 1 | - | - | - | - | 44,9 | 4 | cy |
| 47 | DKFZp434C2415 | AL136757 | 79876 | UBE1DC1 | ubiquitin-activating enzyme E1-domain containing 1 | - | - | - | 2 | - | - | - | - | 1 | 1 | 1 | - | - | - | - | 44,9 | 4 | cy |
| 48 | DKFZp434E1315 | AL133049 | 25896 | INTS7 | integrator complex subunit 7 | - | - | - | - | - | - | - | - | - | - | - | - | - | - | - | 84,3 | 7 | go |
| 49 | DKFZp434E1315 | AL133049 | 25896 | INTS7 | integrator complex subunit 7 | - | - | - | - | - | - | - | - | - | - | - | - | - | - | - | 84,3 | 7 | go |
| 50 | DKFZp434G1415 | AL136759 | 83448 | PUS7L | pseudouridylate synthase 7 homolog (S. cerevisiae)-like | - | - | - | 1 | - | - | - | - | - | - | - | - | - | - | - | 80,7 | 7 | nu |
| 51 | DKFZp434G1415 | AL136759 | 83448 | PUS7L | pseudouridylate synthase 7 homolog (S. cerevisiae)-like | - | - | - | 3 | - | - | - | - | - | - | - | - | - | 1 | - | 80,7 | 7 | nu |
| 52 | DKFZp434I0515 | AL136761 | 81492 | RSHL1 | radial spokehead-like 1 | - | - | - | - | - | - | - | - | - | - | - | - | - | - | - | 80,9 | 4 | cy,nu |
| 53 | DKFZp434I0515 | AL136761 | 81492 | RSHL1 | radial spokehead-like 1 | - | - | - | - | - | - | - | - | - | - | - | - | - | - | - | 80,9 | 4 | cy,nu |
| 54 | DKFZp434I1116 | AL136769 | 56987 | BBX | bobby sox homolog (Drosophila) | - | - | - | - | - | - | - | - | - | - | - | - | - | - | - | 101,6 | 8 | uk |
| 55 | DKFZp434I1116 | AL136769 | 56987 | BBX | bobby sox homolog (Drosophila) | - | - | - | - | - | - | - | - | - | - | - | - | - | - | - | 101,6 | 8 | uk |
| 56 | DKFZp434I0916 | AL136772 | 23213 | SULF1 | sulfatase 1 | - | 1 | 1 | - | - | - | - | - | - | 1 | - | - | 2 | 3 | 3 | 0,9 | 5 | cy,nu |
| 57 | DKFZp434B0417 | AL133067 | 29951 | PDZRN4 | PDZ domain containing RING finger 4 | - | 1 | - | 3 | - | - | - | - | - | 1 | - | - | - | - | - | 18,0 | 11 | nu,nl |
| 58 | DKFZp434I2117 | AL136777 | 83723 | FAM57B | family with sequence similarity 57, member B | - | - | - | - | - | - | - | - | - | - | - | - | - | - | - | 25,2 | 8 | er |
| 59 | DKFZp434F1919 | AL136791 | 60492 | CCDC90B | coiled-coil domain containing 90B | - | - | - | 2 | - | - | - | - | - | - | - | - | 1 | - | - | 29,5 | 7 | mi |
| 60 | DKFZp434G131 | AL136795 | 83449 | PMFBP1 | polyamine modulated factor 1 binding protein 1 | - | - | - | 1 | - | - | - | - | - | - | - | - | - | - | - | 117,5 | 6 | uk |
| 61 | DKFZp434G131 | AL136795 | 83449 | PMFBP1 | polyamine modulated factor 1 binding protein 1 | - | - | - | - | - | - | - | - | - | - | - | - | - | - | - | 117,5 | 6 | uk |
| 62 | DKFZp434D0421 | AL136804 | 81554 | WBSCR16 | Williams-Beuren syndrome chromosome region 16 | - | - | - | - | - | - | - | - | - | - | - | - | - | - | - | 50,0 | 8 | mi |
| 63 | DKFZp434D0421 | AL136804 | 81554 | WBSCR16 | Williams-Beuren syndrome chromosome region 16 | - | - | - | - | - | - | - | - | - | - | - | - | - | - | - | 50,0 | 8 | mi |
| 64 | DKFZp434J1521 | AL136805 | 57616 | TSHZ3 | teashirt family zinc finger 3 | - | - | - | - | - | - | - | - | - | - | - | - | - | - | - | 98,5 | 8 | mi |
| 65 | DKFZp434J1521 | AL136805 | 57616 | TSHZ3 | teashirt family zinc finger 3 | - | - | - | - | - | - | - | - | - | - | - | - | - | - | - | 98,5 | 8 | mi |
| 66 | DKFZp434C2322 | AL136809 | 11093 | ADAMTS13 | ADAM metallopeptidase with thrombospondin type 1 motif, 13 | - | - | - | - | - | - | - | - | - | - | - | - | - | 1 | - | 24,5 | 8 | cy,nu |
| 67 | DKFZp434C2322 | AL136809 | 11093 | ADAMTS13 | ADAM metallopeptidase with thrombospondin type 1 motif, 13 | - | - | - | - | - | - | - | - | - | - | - | - | - | 1 | - | 24,5 | 8 | cy,nu |
| 68 | DKFZp434D0127 | AL136825 | 84101 | USP44 | ubiquitin specific peptidase 44 | - | - | - | 3 | - | - | - | - | - | - | - | - | - | - | - | 81,2 | 7 | nu |
| 69 | DKFZp434P2235 | AL136860 | 84218 | TBC1D3 | TBC1 domain family, member 3 | - | - | - | 2 | - | - | - | - | - | - | - | - | - | - | - | 62,2 | 9 | pm |
| 70 | DKFZp434P2235 | AL136860 | 84218 | TBC1D3 | TBC1 domain family, member 3 | - | - | - | - | - | - | - | - | - | - | - | - | - | - | - | 62,2 | 9 | pm |
| 71 | DKFZp434G099 | AL136904 | 83452 | RAB33B | RAB33B, member RAS oncogene family | - | - | - | 2 | - | - | 2 | 2 | 2 | - | - | - | 1 | 2 | 1 | 25,7 | 7 | go |
| 72 | DKFZp586G2219 | AL136917 | 7286 | TUFT1 | tuftelin 1 | 2 | 3 | - | 2 | - | - | - | - | - | - | - | - | - | - | - | 44,3 | 5 | cy |
| 73 | DKFZp586E1222 | AL136930 | 29097 | CNIH4 | cornichon homolog 4 (Drosophila) | - | - | - | - | - | - | - | - | - | - | - | - | - | - | - | 10,6 | 4 | er |
| 74 | DKFZp586E1323 | AL136936 | 26353 | HSPB8 | heat shock 22kDa protein 8 | - | - | - | 3 | 2 | - | 1 | 2 | 1 | - | 1 | - | 1 | 2 | 2 | 21,6 | 4 | cy,nu |
| 75 | DKFZp586B0424 | AL136940 | 29062 | HSPC049 | HSPC049 protein | - | - | - | - | - | - | - | - | - | - | - | - | - | - | - | 77,2 | 6 | cy |
| 76 | DKFZp586B0424 | AL136940 | 29062 | HSPC049 | HSPC049 protein | - | 1 | 2 | - | - | - | - | - | - | - | - | - | - | - | - | 77,2 | 6 | cy |
| 77 | DKFZp586J0624 | AL136944 | 30061 | SLC40A1 | solute carrier family 40 (iron-regulated transporter), member 1 | - | - | - | - | - | - | - | - | - | - | - | - | - | - | - | 62,5 | 6 | pm |
| 78 | DKFZp586J0624 | AL136944 | 30061 | SLC40A1 | solute carrier family 40 (iron-regulated transporter), member 1 | - | - | - | - | - | - | - | - | - | - | - | - | - | - | - | 62,5 | 6 | pm |
| 79 | DKFZp564H1122 | AL136605 | 28970 | C11orf54 | chromosome 11 open reading frame 54 | 3 | 3 | 3 | 2 | 2 | - | 2 | 2 | 2 | - | - | - | 3 | 3 | 1 | 35,1 | 6 | nu |
| 80 | DKFZp564H1322 | AL136606 | 56006 | C19orf61 | chromosome 19 open reading frame 61 | - | - | - | 2 | - | - | - | - | - | - | - | - | - | - | - | 57,7 | 7 | cy,nu |
| 81 | DKFZp564I0422 | AL136607 | 83591 | THAP2 | THAP domain containing, apoptosis associated protein 2 | - | - | - | - | - | - | - | - | - | - | - | - | - | - | - | 26,3 | 10 | nu,nl |
| 82 | DKFZp564B1023 | AL136611 | 83479 | DDX59 | DEAD (Asp-Glu-Ala-Asp) box polypeptide 59 | - | - | - | - | - | - | - | - | - | - | - | - | - | - | - | 64,6 | 6 | nu- |
| 83 | DKFZp564I0123 | AL136615 | 8575 | PRKRA | protein kinase, interferon-inducible double stranded RNA dependent activator | - | - | - | 3 | 2 | - | - | - | - | 2 | 2 | - | - | 2 | 1 | 34,4 | 8 | cy |
| 84 | DKFZp564A202 | AL080056 | 4714 | NDUFB8 | NADH dehydrogenase (ubiquinone) 1 beta subcomplex, 8, 19kDa | - | - | - | 3 | - | - | - | - | - | - | - | - | - | - | - | 21,7 | 6 | er |
| 85 | DKFZp564D152 | AL136629 | 7259 | TSPYL1 | TSPY-like 1 | 2 | 1 | 2 | 3 | 2 | 2 | 1 | 2 | 1 | - | - | - | 2 | 2 | 1 | 49,4 | 5 | nu |
| 86 | DKFZp564G182 | AL136632 | 81688 | C6orf62 | chromosome 6 open reading frame 62 | 2 | 3 | 3 | 2 | 2 | - | 2 | 2 | 2 | - | - | - | - | 1 | 1 | 27,1 | 9 | cy,nu |
| 87 | DKFZp564H012 | AL136633 | 29074 | MRPL18 | mitochondrial ribosomal protein L18 | - | - | - | - | - | - | - | - | - | - | - | - | 1 | - | 1 | 20,6 | 10 | mi |
| 88 | DKFZp564M082 | AL080071 | 25906 | C11orf51 | chromosome 11 open reading frame 51 | - | - | - | - | - | - | 2 | - | 2 | - | - | - | - | - | - | 14,3 | 3 | cy,nu |
| 89 | DKFZp564C1362 | AL136647 | 10229 | COQ7 | coenzyme Q7 homolog, ubiquinone (yeast) | - | - | - | 3 | - | - | - | - | - | 2 | - | - | - | 1 | 1 | 20,3 | 5 | uk |
| 90 | DKFZp564A1164 | AL136654 | 84063 | KIRREL2 | kin of IRRE like 2 (Drosophila) | - | - | - | - | - | - | - | - | - | - | - | - | - | 1 | - | 21,3 | 6 | cy,nu |
| 91 | DKFZp564K2478 | AL136690 | 11274 | USP18 | ubiquitin specific peptidase 18 | - | - | - | - | - | - | - | - | - | - | - | - | - | - | - | 43,0 | 7 | nu |
| 92 | DKFZp564N0582 | AL050264 | 11170 | FAM107A | family with sequence similarity 107, member A | 2 | 2 | 3 | 3 | 3 | 2 | 2 | 2 | 3 | - | - | - | 2 | 2 | 1 | 17,5 | 10 | uk |
| 93 | DKFZp566K144 | AL136727 | 84084 | RAB6C | RAB6C, member RAS oncogene family | - | - | - | - | - | - | - | - | - | - | - | - | - | 1 | - | 23,3 | 6 | go |
| 94 | DKFZp434P1735 | AL136859 | 55130 | ARMC4 | armadillo repeat containing 4 | - | - | - | - | - | - | - | - | - | - | - | - | - | - | - | 0,6 | 3 | mi |
| 95 | DKFZp434J0450 | AL136869 | 9400 | RECQL5 | RecQ protein-like 5 | - | - | - | 2 | - | - | 3 | 3 | 3 | - | - | - | 2 | 2 | 2 | 19,3 | 10 | nu |
| 96 | DKFZp434E248 | AL136897 | 55341 | LSG1 | large subunit GTPase 1 homolog (S. cerevisiae) | - | - | - | 2 | 1 | - | - | - | - | - | - | - |  | - | - | 75,2 | 6 | er |

^a^ Additional information on the encoded proteins are accessible via [http://www.LIFEdb.de](http://www.LIFEdb.de/)

^b^ Accession number according to GenBank entry

^c^ NCBI/Entrez gene symbol and gene ID

^d^ Protein name according to GenBank entry

^e^ Protein yield presented for the induction temperatures of 25°C, 30°C, 37°C. Numbering 1, 2, and 3 gives the apparent yield and concentration of each fusion protein. 1: 0.5 - 2.5 µg/mL; 2: 2.5 - 10 µg/mL; and 3: >10 µg/mL. Yield was estimated by comparison to BSA standards after Coomassie staining of SDS-gels. The preparation of entry clones failed for ORF no. 30, 31, 32, and the preparation of expression clones for ORF no. 20, 62

^f^ MW refers to the size of respective proteins without tag.

^g^ pI is the isoelectric point of the protein without tag.

^h^ Subcellular localizations are abbreviated as follows: cy, nu: cytoplasm and nucleus; ne: nucleus-envelope; nu: nucleus; go: Golgi; mi: mitochondria; cs, mt: cytoskeleton-microtubules; er: endoplasmatic reticulum; cy: cytoplasm; nu, nl: nucleus-nucleolus; pm: plasma membrane; or: others, uk: unknown
